# The relationship between emotional regulation and hemispheric lateralization in depression: a systematic review and a meta-analysis

**DOI:** 10.1038/s41398-022-01927-9

**Published:** 2022-04-16

**Authors:** Natia Horato, Laiana A. Quagliato, Antonio E. Nardi

**Affiliations:** Instituto de Psiquiatria, Universidade Federal do Rio de Janeiro, Rio de Janeiro, Brazil

**Keywords:** Biomarkers, Physiology

## Abstract

From a neurobiological perspective, diverse studies have associated emotional regulation with cognitive deficits. Structural and/or metabolic changes in the frontal cortex are often inferred from dysfunction in cognitive-emotional processing. In addition, electroencephalographic findings support the idea that alpha band oscillations are responses to these same processes. Thus, the objective of this meta-analytical literature review is to verify whether the possible hemispheric lateralization attributed to frontal alpha asymmetry (FAA) correlates with emotional regulation and the cognitive deficits underlying depression. The data included in our meta-analysis are from articles published from 2009 to July 2020, which utilized DSM or ICD criteria to diagnose depression or anxiety disorders and included a control group. For statistical analysis, the measurements obtained through the 10–20 electroencephalography system were used. The frontal alpha asymmetry index was calculated from the difference between the logarithm of the absolute spectral values in the alpha rhythm observed from the F4 and F3 electrodes that were fixed to the scalp of the frontal region of the right and left hemispheres (ln µV² RH−ln µV² LH) = (F4−F3). Eighteen articles were included in the systematic review. Of these, 9 were homogeneous enough for statistical analyses (total *N*: 1061; *N*_Dep_: 326; *N*_cont_: 735). Nine others could not be statistically analyzed due to the absence of FAA measurements from the F4 and F3 electrodes. A random effects meta-analysis revealed low heterogeneity (Qt = 11,00*,* d*f* = 8, *p* = 0.20, *I*^2^ = 27%) and an average effect size of the studies equal to −0.03 (CI = [−0.07 to 0.01]). The results, although not significant, suggested a slight tendency toward left lateralization in the depression group. Although the effects shown in these data did not confirm hemispherical lateralization in depressed patients, it was found that emotional regulation and cognitive processes share similar neural circuits. Therefore, future research on this complex relationship is encouraged, especially studies that are focused on the search for quantitative biological markers in depression.

## Introduction

Emotional regulation refers to the ability to manage our behavioral response in the face of challenging and stressful everyday situations. It is a complex process responsible for starting or inhibiting reactions triggered by various stimuli. However, individual levels of affective traits and structural changes in specific regions of the brain have been associated with the ability to regulate emotions [1]. The persistence of negative affect [2], differences in hemispheric brain activation and changes in functions needed for executive control are characteristics that have been verified in some psychiatric disorders [3]. However, depressive disorder (DD) and anxiety disorders (AD) share many symptoms associated with cognitive deficits and emotional regulation [4]. Both anhedonia, a striking feature in depression [5], and the impulsivity present in generalized anxiety disorder [6] seem to be a consequence of the increase in negative emotions, suggesting that the neurobiology of emotional regulation is fundamental to the psychopathology.

Patients with anxiety disorder often have depressive symptoms and vice versa [7]. The investigation of similarities and differences between depression and anxiety, points to patterns of similar neural responses, especially in the frontal region of the brain [8]. In addition, some studies reinforce that the dysregulation of pathophysiological mechanisms, such as the neurotransmitter systems norepinephrine and serotonin, interfere with the symptoms of depression and anxiety [9]. And that the elevation of secretion of the corticotrophine-releasing hormone is recurrent in these disorders [10]. Through electroencephalography, it is also possible to observe that anxious and depressive patients present cortical activity dysfunction in analogous brain regions [11]. Thus, recent studies suggest that these disorders are highly comorbid [12].

Among the brain structures that possibly participate in emotional regulation strategies, the importance of the dorsolateral prefrontal cortex (dlPFC) has been emphasized because the behavioral flexibility of individuals who suffer damage in this region is compromised evidenced by difficulties observed in performing tasks involving working memory and decision-making [13]. In addition, correlates of neural dysfunctions, such as indecision, difficulty concentrating, prostration and lack of perspective for the future, may be observed among patients with symptoms of depression and patients with left dlPFC injury [3]. This corroborates our hypothesis that this brain region is involved in cognitive and emotional processes. In addition, electroencephalographic findings support the idea that alpha band oscillations (8–13 Hz) in the right and left dlPFC are related to these same processes [3].

In line with these findings, some studies have sought ways to measure the activity of the dlPFC in depressive conditions using EEG as a tool for data collection. Through the difference between the logarithm of the absolute spectral values in the alpha band, in the electrodes located in the frontal region of the right and left hemispheres (ln µV² RH–ln µV² LH), the frontal alpha asymmetry index (FAA) is obtained, where alpha potency is inversely proportional to cortical activity and negative values suggest left hypoactivation, while positive values indicate right hypoactivation [14]. Although FAA is susceptible to variation, it has been widely disseminated as a possible biological marker in depression [15].

It has been shown that the decrease in the severity of depressive symptoms corresponds to increased dorsolateral cortical activity [16] and that cognitive ability derives from cortical activation of this same region [17]. In addition, it was observed that the power of the alpha rhythm was negatively correlated with cortical activity in the dlPFC [14]. Since all these aspects are intrinsic to the dlPFC, the objective of this study was to relate emotional regulation to hemispheric lateralization in depression and to offer a meta-analysis that verifies the existence of hemispheric lateralization attributed to the FAA. We calculated the effect size for nine studies that evaluated oscillations in the alpha band [15, 18–25], using FAA with reference to electrodes F4 and F3 and their respective standard deviations as EEG measures for the comparison of subjects who comprised the depression group versus participants in the control group.

## Methods

A systematic search was conducted in the PubMed and Cochrane databases, covering articles published from 2009 until February 2020. The search protocol was developed based on Preferred Reporting Items for Systematic Reviews and Meta-Analyses (PRISMA), and it was previously registered in PROSPERO (CRD42020150815) [26]. To avoid publication bias, non-English language studies and those in the gray literature (for example, conference abstracts) were included. A broad but highly structured search strategy was used based on the PICOS framework. The study population was subjects with a depressive or anxiety disorder, the intervention/exposure was EEG measurements, the comparison was to a control group, the outcome was the evaluated hemispheric lateralization attributed to the FAA and the study design included any type of design.

A search for articles published from 2009 in the PubMed and Cochrane databases was performed using the following keywords: executive function OR executive control OR cognition OR prefrontal cortex OR dorsolateral prefrontal cortex OR dlPFC activity OR lobe OR cerebral cortex OR cerebral mantle OR pallium OR prefrontal lobe OR cortex OR brain imaging OR EEG OR neuroimaging OR cognitive reappraisal OR working memory OR inhibitory control OR emotional regulation OR decision making OR cognitive reevaluation AND emotion OR sadness OR prostration OR melancholy OR heartbreak OR adversity OR distress OR anguish OR bitterness OR suffering OR euphoria OR joy OR happiness OR pleasure OR satisfaction OR impulsiveness OR contentment OR behavior OR self-control OR attention OR stress OR nervousness OR frenzy OR mood OR disorder OR anxiety OR depression OR irritability AND anxiety disorder OR generalized anxiety disorder OR panic disorder OR mood disorders OR depressive disorder OR separation anxiety disorder OR agoraphobia OR phobia AND brain imaging OR neuroimaging electroencephalogram.

The following data were collected: year of publication, first author, number of participants, age, mental disorder, EEG measurements, and standard deviation. In the statistical analysis of the data, only the following variables were used: number of participants, EEG measure (international system 10−20), and standard deviation.

For the EEG measurement, we considered the frontal alpha asymmetry index (FAA), calculated from the difference between the logarithm of the absolute spectral values in the alpha rhythm, observed from the F4 and F3 electrodes, fixed to the scalp of the frontal region of the right and left hemispheres (ln µV² RH–ln µV² LH) = (F4−F3). There was no standard deviation imputation for any article. Only 1 article had the standard deviation calculated, where the equation was Sd = (*P*/*t*)*N group (*P* = difference between means; *t* = test; *N* group = number of participants from *each* group).

Statistical analyses of the variables were carried out with the aid of RevMan 5, Cochrane software. Using a random effects model and observing the mean differences between the intervention and control groups, we consider the 95% confidence interval for the obtained results. The heterogeneity was attributed to the different sample sizes among the groups of participants in the selected studies.

## Results

**Fig. 1:**
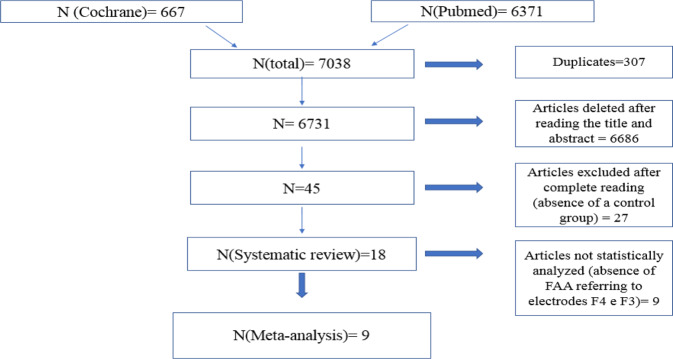
PRISMA 2020 Flow Diagram. Studies included in the review. The meta-analysis was performed according to the PRISMA Guidelines.

**Fig. 2:**
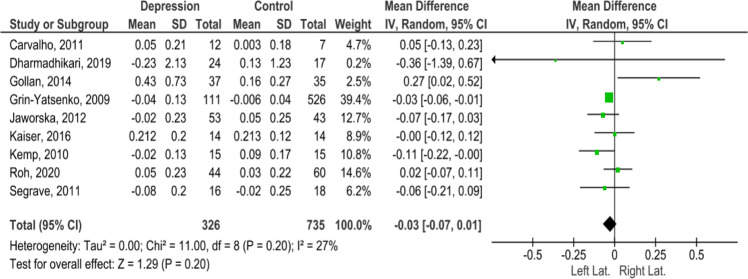
Forest plot. Hemispheric lateralization in depression. The effects shown in these data did not confirm hemispheric lateralization in depressed patients.

A total of 7038 potential articles for initial screening were published as of 2009. Of this total, 307 duplicates were identified through the Mendeley reference manager and with manual confirmation of the titles. After the exclusion of duplicates, 6731 articles remained, of which 6686 were rejected based on the reading of titles and abstracts and 45 were selected for the full reading of the texts. Then, 27 other studies were excluded because they did not meet the eligibility criteria. Therefore, 18 articles were included in this review. Of these, 9 were homogeneous enough for meta-analysis (Fig. 1). Another 9 studies could not be statistically analyzed due to the absence of the alpha band frontal asymmetry index (FAA) measure for electrodes F4 and F3 (Fig. 2).

Articles that did not present FAA measurements related to F4 and F3 electrodes were excluded from the meta-analysis during data extraction (Table 1). To minimize the heterogeneity among the studies included in the analysis, for the studies with FAA measurements in different regions and brain waves, only the measurements referring to the alpha wave in the frontal region were collected. However, specifically from the F4 and F3 electrodes, as already mentioned earlier in the topic methodology (Table 2).

**Table 1:** Summary of studies included in the systematic review.

Study	Diagnosis	Total sample (%)		Intervention group		Control group		EEG measure	Rhythm (Hz)
		Female	Male	Subjects	Mean age	Subjects	Mean age		
1 Begić et al., 2011	Depression	68	32	33	55.1	30	35.9	ASP	Delta, Theta, Alpha, Beta
2 Cook et al., 2014	Depression	41	59	121	40.6	47	37.9	ASP/RSP	Theta
3 Hinrikus et al., 2009	Depression	100	0	18	35	18	35	SASI	Theta, Alpha, Beta
4 Iseger et al, 2017	Depression	58	42	1008	38	336	37	ASP	Theta, Alpha
5 Kan et al, 2015	Depression	37.5	62.5	4	23.3	4	23.3	ASP	Alpha
6 Liu et al., 2019	Anxiety	55	45	40	19.8	40	19.5	ASP	Beta
7 Olbrich et al., 2014	Depression	100	0	60	39.4	60	37.6	ASP	Delta, Theta, Alpha, Beta
8 Li et al., 2017	Depression	55.5	45	33	32.8	32	29.5	PSI	Delta, Theta, Alpha, Beta
9 Wang et al, 2016	Anxiety	53	47	64	46.41	30	45.41	D2	Theta, Alpha, Beta

*ASP* absolute spectral power values, *RSP* relative spectral power, *SASI* spectral asymmetry index, *PSI* phase synchronization index, *D*2 correlation dimension.

**Table 2:** Summary of studies included in the meta-analysis.

Study	Total sample (%)		Diagnosis measure	Subj.	Depression group			Control group				Alpha (Hz)	Eye
	Female	Male			Mean age	FAA	SD	Subj.	Mean age	FAA	SD		
1 Carvalho et al. (2011)	63	37	BDI	12	71	0.05	0.21	7	72	0.003	0.18	8–13	C
2 Dharmadhikari et al. (2019)	63	37	HRSD	24	34.8	−0.23	2.13	17	29.5	0.13	1.23	8–13	O/C
3 Gollan et al. (2014)	62.5	37.5	IDS-C	37	35.6	0.43	0.73	35	35.6	0.16	0.27	8–13	O/C
4 Grin-Yatsenko et al. (2009)	58	42	DSM-IV	111	38.5	−0.04	0.13	526	35.1	−0.006	0.04	7.5–14	O/C
5 Jaworska et al. (2012)	54	46	HRSD	53	40.4	−0.02	0.23	43	36.5	0.05	0.25	10–12	C
6 Kaiser et al. (2016)	100	0	HRSD	14	80.5	0.212	0.20	14	78.6	0.213	0.12	8–13	C
7 Kemp et al. (2010)	60	40	HRSD	15	39.9	−0.02	0.13	15	42.4	0.09	0.17	8–13	O/C
8 Roh et al. (2020)	85.5	14.5	HRSD	44	38.3	0.05	0.23	60	34.8	0.03	0.22	8–13	O/C
9 Segrave et al. (2011)	100	0	BDI	16	42.1	−0.08	0.20	18	40.7	−0.02	0.25	8–13	O/C

Reference (Cz); FAA, F4−F3; *C* close, *O* open.

## Systematic review

All studies that were part of this literature review investigated how brainwave activity was associated with emotional regulation and human cognition. By using EEG measurements in their experiments, it was possible to verify whether brainwave oscillations would be a potential biomarker in depression and anxiety when compared to the control group [27].

Researchers have sought to understand the cause of these oscillations, especially in the frontal and prefrontal regions, in individuals with symptoms of depression [28, 29]. In anxiety disorders, significant oscillations of cortical activity have been observed in all brain regions, especially in the areas in the left hemisphere [30]

Highly concerned individuals tended to show greater changes in the beta band in multiple brain regions [31]. In depressive subjects, beta oscillations are concentrated in the central frontoparietal region, which would explain the attention deficit proportional to the severity of symptoms [32].

The studies demonstrated similar results regarding oscillations in different brain waves; however, there are specificities to consider: Iseger [33], for example, stated that alpha band changes can serve as a potential biomarker in the treatment of depression, but only in men. In addition, the prefrontal cortex presented higher alpha power in individuals monitored only in the open-eyes condition [34]. Schizophrenic patients differed from depressive patients in delta power values, which would favor the correct diagnosis between the two disorders [29].

Although the 9 articles excluded [27–35] contributed significantly to our literature review, restrictions on alpha band FAA measures made it impossible to include them in our statistical analyses.

## Meta-analysis

The studies used in the statistical synthesis investigated the existence of hemispheric lateralization of the alpha wave in depression. The FAA (F4−F3) was calculated from the difference between the logarithms of the absolute spectral values of the alpha wave from the F4 and F3 electrodes. In the assembly of the electrodes, the Cz reference was considered, where F4 was located on the right scalp and F3 was located on the left scalp. For negative FAA, we have the value of F3 > F4, which means higher alpha power in the left front hemisphere (left alpha lateralization) and decrease of cortical activity in this region, as they are inversely proportional magnitudes. In the same way, when FAA is positive, we have that F4 > F3 and alpha power focuses on the right front hemisphere (right alpha side), with consequent reduction of cortical activity on the right [14].

Some studies included in the meta-analysis showed divergent outcomes. This was possibly due to individual characteristics of the study, such as how many participants and the age of the participants. The studies that showed the lowest numbers of participants, i.e., depression group, *N* = 12/control group, *N* = 7 in ref. [18] and depression group, *N* = 14/control group, *N* = 14 in ref. [23], did not show a relationship between frontal alpha asymmetry and depression in elderly individuals. In ref. [21], a study with a higher number of participants (depression group, *N* = 111; mean age = 38.5, and control group, *N* = 526; mean age = 35.1), the decrease in cortical activation was reflected in the patterns of FAA. Thus, alpha asymmetry is a highly variable phenomenon, and the sample size of participants influences the reliability of the results. Therefore, even if there are no statistically significant differences in FAA, further studies are encouraged [15].

Other important individual characteristics are highlighted below: Dharmadhikari [19] suggested FAA as a biological marker in depression but admitted its limitations due to a sample of nonrandomized participants. Gollan [20] observed important asymmetric differences in the alpha wave and associated them with behavioral activation. Frontal alpha asymmetry also indicated hypoactivity in the left frontal hemisphere [22], as well as an overall increase in alpha power in depressive disorders [24]. Patients with severe depression presented higher indices of frontal alpha asymmetry, even in the absence of suicidal ideation [25].

Although our meta-analysis does not demonstrate significant statistical results regarding the role of FAA as a hemispheric lateralization parameter, we observed a slight tendency toward left lateralization in the depression group (Fig. 2).

In the selected studies, there were a total of 1061 participants. Of these, 326 belonged to the depression group, and 735 belonged to the control group. A random effects meta-analysis showed low heterogeneity (Qt = 11.00, d*f* = 8, *p* = 0.20, *I*^2^ = 27%) and an average effect of the studies equal to −0.03 (CI = [−0.07; 0.01]). The heterogeneity, although not significant, was attributed to the different sample sizes of participants in the selected studies (Fig. 2).

## Discussion

This study evaluated the relationship between emotional regulation with hemispheric lateralization in depressive patients. In addition, cognitive deficits underlying depression were described. From a neurobiological perspective, it was found that structural and/or metabolic alterations were associated with dysfunction in cognitive-emotional processing. Recent studies corroborated these findings, stating that cortical hypoactivity observed in lesions specific to the dlPFC has underlying sequelae, such as increased symptoms of depression and cognitive deficit [36].

Attributed to the prefrontal cortex, executive control refers to the human capacity to manipulate information, inhibit behaviors, change the attentional focus, and solve everyday problems, among other competencies [17]. Thus, abnormalities in the neural circuits of this brain region have important consequences on cognition [36]. Curiously, in depressive disorder, there are common clinical complaints about the decline of skills granted to executive control, such as impaired memory and concentration [4]. This suggests that cortical hypoactivity is a common characteristic of cognitive and emotional disorders [3].

Among the neurological disorders that influence cortical activity, we investigated oscillations in the alpha band (8–13 Hz), specifically in the frontal region [3]. Observable in the resting state, without sleep, the alpha wave can be detected and standardized through electroencephalography (EEG). Its unsynchronized wavy rhythm has a negative relationship between amplitude and frequency. Therefore, the higher the alpha power, the lower the cortical activation [36]. Considering the brain patterns responsible for relaxation, greater alpha amplitudes reflect lower capacity for concentration, planning, and decision making [20].

Cognitive skills allow us to control attitudes, thoughts, and emotions. In addition, we verified that in the presence of negative affect, low intentional engagement and changes in alpha rhythm are frequent events [17]. Thus, we understand that any irregularities in executive control will have implications for mood regulation [2].

In an attempt to identify possible numerical parameters in the interaction between emotional regulation and hemispheric lateralization in depression, we used the frontal alpha asymmetry index (FAA) as an investigative instrument. Although previous studies have achieved similar outcomes to those described in this work, the explanation of the correlated aspects between depression and anxiety proposes a reflection on the extent to which shared and overlapping symptoms of anxiety can covariate alpha asymmetry in depression. We believe this is a relevant aspect in our work.

In this meta-analysis, the role of FAA as a supposed predictor of hemispheric lateralization was investigated. Negative values indicate greater alpha amplitude and lower left cortical activity in depressed subjects when compared to the control group. The data collected included adult populations of both sexes, who manifested symptoms of depression, without confirmation of psychiatric comorbidities. Our results were not statistically significant; however, a subtle propensity toward left lateralization was noticed in the depression group. This suggests that individual aspects of a study, such as sample size and age group of the participants, are variables influencing the size of the effect and divergence between the studies [15].

Despite all efforts, the greater sample heterogeneity and variation in method design made it impossible to integrate studies on depression and anxiety into meta-analysis. Therefore, the scientific gaps in the dialectic involving these disorders are still not clear. Thus, this work includes a systematic review with anxious and depressive patients, and a meta-analysis only with depressive patients [37].

As a limitation of our work, the exclusion of FAA obtained in other pairs of electrodes located in the frontal region should be emphasized: Fp1 and Fp2 [13] and F7 and F8 [15, 19–22, 25]. Only the data from F4 and F3 were adopted because they are the most common electrodes in the literature for the analysis of frontal alpha asymmetry. Another limitation was the exclusion of the asymmetries related to delta, theta and beta waves [27, 28, 32, 33, 35]. Our decision to explore only the alpha wave was based on findings that correlated certain cognitive and behavioral characteristics to specific frequency bands. In fact, alpha waves have often been associated with information processing, and alpha wave oscillations have significant implications for executive control [38]. Thus, it is necessary to consider future studies that reconsider the aspects not addressed in this work.

In conclusion, the effect of our study does not confirm hemispherical lateralization in depressed individuals, it was found that emotional regulation and cognitive processes share similar neural circuits. Therefore, we encourage further research on this complex relationship, especially studies dedicated to the search for quantitative biological markers in depression. The increase in biomarker data for depression will represent a concrete and tangible tool, providing greater accuracy in the diagnosis of depressive disorder and ensuring patients an early and adequate treatment.
